# Supplementary material for: Are carbon emissions trading and green financial instruments synergistic? -Comprehensive quantitative research based on content analysis
Source: PLoS One. 2024 Mar 7;19(3):e0298601. doi: 10.1371/journal.pone.0298601 (PMC10919601; doi:10.1371/journal.pone.0298601)
Supplement: S1 Data — (ZIP) [file pone.0298601.s001.zip › Data/地级市Z_F工作报告.zip!/░▓╗╒/╗┤─╧╩╨/└┤╫╘╣1⁄2╓┌║┼í╛┬φ┐╦╩2╛▌═°í┐_2012.docx]

**各位代表：**

      现在，我代表市人民政府，向大会作政府工作报告，请予审议，并请市政协委员和其他列席人员提出意见。

      一、科学发展，赢得“十二五”良好开局

      刚刚过去的2011年，是“十二五”开局之年。全市人民在市委的坚强领导下，按照中央和省委、省政府的决策部署，全面贯彻市第九次党代会精神，积极应对严峻复杂宏观形势，远谋近施，奋力开局，圆满完成市十四届人大五次会议确定的目标任务。
预计，全市生产总值突破700亿元，增长12%以上；财政收入138.85亿元，增长30.9%；规模以上工业增加值460亿元，增长15%；固定资产投资520亿元，增长30%；社会消费品零售总额220亿元，增长18%；进出口总额2.2亿美元，增长65.4%；城镇居民人均可支配收入18000元，增长17%；农民人均纯收入6700元，增长16.5%；单位生产总值能耗下降4%；COD、二氧化硫、氨氮分别削减1.5%、3.3%、1.5%，氮氧化物排放总量控制在省下达指标内。荣获中国招商引资最具投资潜力城市、最佳投资城市、最具幸福感城市、最具魅力节庆城市，被联合国环境规划署授予“中国区环境规划示范城市优秀案例”奖。
（一）重大项目和转型发展实现新突破。围绕实施“十二五”规划，强力推动项目建设。项目谋划与推进、规划生成项目、月度集中开工、“双十”重大工程等重要举措扎实推进，全年实施项目1080个，增长32.6%。朱集矿、顾桥煤矸石电厂等基本建成，朱集西矿、1000千伏特高压获得核准，淮沪二期、淮浙二期、平电三期获批开展前期工作。园区建设取得新突破。修编完成园区中���期发展规划，出台加快园区建设和发展若干优惠政策意见。市经济技术开发区20平方公里扩区工作全面完成。陕汽新能源汽车一期、舜立机械、凯盛重工、北新建材、国瑞药业等项目开工建设。高新区开发全面启动，国家煤化工产品质检中心竣工，日芯光伏一期等项目加快推进，大气环境科技产业园等项目开工。煤化工产业园功能加快完善，煤化一体化项目推进有力，煤制天然气项目获国家批准。建设园区标准化厂房40万平方米。40户企业加快退城进园。省级园区工业增加值增长30%。产业培育取得新突破。工业、服务业、战略性新兴产业“十二五”发展规划编制完成。“千百十”工程强力实施。“223”工程加快推进，248个项目列入省技改计划。全年新增规模以上工业企业40家。煤机装备制造业集聚效应凸显。比塞洛斯煤机、长壁煤机达产增效。商贸物流业加快发展。龙湖路地下商业街、淮矿物流园、安成鑫海物流园等建成投用。文化旅游产业取得新成果。《淮南子传奇》荣获中国动漫最高奖“美猴奖”、国家动漫精品工程创意奖。龙湖景区获评国家4A级景区，新增国家3A级景区2处。战略性新兴产业实现产值39.5亿元，增长41%。高新技术产业实现增加值18.5亿元，增长28%。创新能力建设取得新突破。荣膺全国科技进步先进市、质量兴省先进市。潘集区被批准为省级可持续发展实验区。国家防爆电气优质产品生产示范区创建扎实推进。国内首个“云海战略”创新实践区启动建设。获批国家创新和成果转化项目17个，认定国家火炬计划重点高新技术企业、国家级企业技术中心各1家。参与起草国家、行业技术标准5项。专利申请1800件，授权680件。新增省著名商标6件。“美亚”牌锚固剂荣获中国驰名商标。节能减排取得新突破。实施重点企业效能对标活动，新增重点节能项目30个，宏泰钢铁技改等项目竣工，淘汰落后炼焦产能30.6万吨。火电机组脱硝、机动车污染防治等工作扎实开展。西部和凤台污水处理厂提浓提量，山南污水处理厂、6座生态式污水处理站、1.6万座无害化厕所全面建成。创建省级生态乡镇4个。跻身国家农村环境连片整治示范市。

     （二）对外开放和深化改革开创新局面。坚持上下联动，强力实施“招商引资年”活动。招商引资成效显著。组团参加“9·8”投洽会、中博会等八大节会，扎实开展赴欧美、台湾等地招商，全年共签约项目223个。央企、知名民企、皖粤经贸合作项目累计开工116个。全年引进内资430亿元，增长65.4%；利用外资1.6亿美元，增长20.5%。中国豆腐文化节、国际少儿艺术节品牌效应日益彰显，获得最具创新价值节庆、中国十大节庆产业金手指奖。交流合作成果丰硕。成功举办合肥经济圈城市第二次会商会，发表《淮南宣言》，签署4项框架协议。积极申请加入长三角经济协调会，大力承接产业转移。加强与国内7家科研院所和企业战略合作，提升创新成果孵化水平。加强与德国多特蒙德市、泰国正大集团战略合作，与美国帝国郡签订友好城市备忘录。加强与美国安科、瑞孚乐公司技术合作，推动世界一流的高倍聚光太阳能光伏、透镜生产技术和产品落户。重点改革成功推进。积极争取纳入国家资源型城市可持续发展规划。市招投标监督管理局挂牌运行。启动设立海关和出入境检验检疫局申报工作。在地统计制度改革扎实推进。农业、教育投资公司挂牌成立，城投、产投、旅游发展公司改革步伐加快。基本完成国有企业“出中心”职工债权清偿工作。6户企业签订上市辅导协议。非煤矿山企业由14户整合到7户。银企对接资金92.2亿元。引进市外金融机构3家，新开业小额贷款公司4家，新批准融资性担保机构3家，在全省率先建成中小企业信用信息平台。新增民营企业1710户、个体工商户9760户。

       （三）城市建设和规划管理展示新风貌。城市化率达63%，提高1.2个百分点。大建设三年投融资计划顺利实现，全年完成大建设投资310亿元，增长40.3%。城市形象日益彰显。“五彩淮南”形象宣传片在央视播出，城市知名度和美誉度大幅提升。新区开发提速。淮南二中、人防教育基地等15个项目主体工程竣工。正源大厦、市档案馆新馆、五星级酒店等18个项目加快推进。市会议中心、居民安置点等27个项目开工建设。老区建设提质。滨河滨湖新区前期工作稳步推进。开工老城区综合改造项目16个，整治城市旧小区16个、小街小巷95条。矿区改造提量。开工棚户区改造住房15158套。景区完善提效。龙湖景区二期、八公山文化中心、焦岗湖影视城一期基本建成，泰坦方舟动漫园、淮河公园等12个项目加快推进。综合交通提升。东西部第二通道、206国道改线全线贯通，淮潘公路升级改造、淮河大桥维修加固、惠利大道改扩建等一批工程完工。京福高铁淮南段暨淮南东站、淮蚌高速公路加快建设，朝阳东路延伸等6个新建工程、合淮路改扩建等工程扎实推进。凤台二桥正式开工。山南新区智能交通管控体系基本建立。山南七乡镇公交线路开通。城市品位日益提升。城市轨道交通等7个专项规划、滨河新区等16个控制性详细规划编制完成，村镇规划加快推进，“新汉风”建筑与规划深入研究。数字城管一期建成，城区道路标识牌全面更新，城管志愿者活动扎实开展，人性化、市场化管理机制初步建立。加大城市管理投入，新增环卫洗扫设备59台，机械化作业水平明显提升。完成成片造林1.38万亩，新增城市绿地100万平方米，拆除违法建设30万平方米。全国绿化模范城、国家生态园林城创建扎实推进，荣获创建省级文明城市工作先进市。

       （四）现代农业和城乡一体取得新成效。全市“三农”支出21亿元，增长15%。现代农业大力发展。出台创建稻麦“吨粮市”实施意见、进一步加强“菜篮子”工程建设意见。全年粮食总产141.6万吨，连续8年喜获丰收，凤台县荣获全国粮食生产先进县。在全省率先建立市县级粮油储备机制。蔬菜、肉蛋奶、水产品产量均实现增长。认证“三品”25个。新增全国绿色食品原料生产基地、国家级农业标准化示范区各1个。新增省级农业产业化龙头企业5家、省级以上“放心粮油”示范企业15家、农民专业合作社114家。农业产业化总产值130亿元，增长26%。新农村建设大力推进。10个城乡一体化示范乡镇、50个示范村、44个新农村建设项目扎实开展，农村清洁工程示范村扩大到300个。建成“万村千乡”市场工程配送中心13个，农家店实现乡级全覆盖。完成标准化菜市场改造32个。启动9条县乡道路升级改造，建成5个农村客运站。完成农村危桥改造19座。整村推进10个土地整治项目，新增耕地1.1万亩、土地流转9.8万亩、退网还湖2.7万亩。出台加快水利改革发展实施意见。禹王泵站更新改造、泥河疏浚、3座水库除险加固等工程基本完成，淮河重点平原洼地治理项目全面实施，永幸河站重建、乔口站新建工程开工建设。防汛抗旱成效显著，夺取了“8·26”凤台特大暴雨救灾的胜利。

       （五）民生改善和社会事业得到新提升。全年投入资金15.4亿元，深入实施42项民生工程。住房公积金贷款支持保障性住房建设试点工作有序开展。建成公租廉租住房1407套，发放廉租住房补贴3226万元。完成农房改造1755户。解决农村3.5万人安全饮水问题。完成校舍安全工程53.3万平方米。三项医疗保险参保人数达229.8万人。基层医药卫生体制综合改革全面深化，凤台县公立医院改革试点扎实推进，公益性管理体制基本建立。创建三级甲等、三级乙等医院各1所。市120指挥中心、医疗废物处置中心建成投用。基层卫生服务站所建设提前两年完成。省市共建城乡一体化食品药品安全示范市正式启动，乡镇以上医疗机构规范药房建设全部达标。“数字物价”工程开工建设，建立困难群体救助标准和物价上涨联动机制。城镇职工五项社会保险参保达159万人次。新型农村养老保险逐步推进。城乡低保和农村五保供养实现扩面提标。市社会福利院综合楼、市流浪未成年人救助中心、市光荣院开工建设。全年投入资金15.1亿元，大力推进采煤塌陷区综合治理。新开工安置点13个、278.7万平方米，竣工10个，搬迁安置居民4.4万人。实施塌陷区环境综合治理项目107个。全年支出资金80.3亿元，加快发展社会事业。省级创业型城市加快建设，建成农民工创业园9个、大学生创业孵化基地1个。新增城镇就业岗位5万个，转移农村劳动力3.9万人，城镇登记失业率4.1%。安徽理工大学新校区、淮南文化艺术中心开工建设，市职教中心获批筹建国家中职示范学校，凤台、潘集职教中心建成投用。文化服务体系得到完善，少儿艺术跻身首批国家公共文化服务体系示范项目。政府机关软件正版化淮南模式在全国推广。全民健身运动蓬勃开展。成功举办全国人口计生工作现场会。人口自然增长率7.5‰。民族团结、宗教和睦局面继续巩固，国防动员、人民防空水平不断提升。外事、侨务、地震、气象、档案、地方志工作持续加强，妇女、儿童、老龄、残疾人事业扎实推进。

       （六）管理创新和环境优化彰显新形象。自觉接受法律监督、民主监督、舆论监督和社会监督，认真汲取各方面意见和建议。全年办理人大代表议案建议129件，政协委员提案403件。启动“六五”普法规划，深入开展“江淮普法行”活动。加强政府立法，提请审议地方性法规2件，制定政府规章4件。办理行政复议和应诉案件50件。法律援助1800余件，调处矛盾纠纷1万余起。深化政务公开，健全网络问政机制，网民留言回复率达99%。出台进一步优化投资环境若干意见，行政审批提前办结率达92%。圆满完成第八届村委会换届选举工作。扎实推进“安全生产年”活动。成功举办全国煤矿瓦斯防治现场会，淮南经验在全国推广。淮河流域突发环境事件应急演练取得圆满成功。社会稳定风险评估机制全面落实，重大突发事件应急处置机制和社区网格化管理服务新模式初步建立。妥善处置涉疆“1·20”等事件，受到中央领导肯定。公安“三项建设、三项重点工作”扎实推进。深入开展大接访、大走访和千案攻坚专项活动，治安大局持续平稳，社会保持和谐稳定。党风廉政建设责任制严格落实，惩治和预防腐败体系建设深入推进，民主评议市直机关科处室负责人活动有效开展。建立重大项目监察督查机制，严厉查处违法违纪案件。从严控制“三公”消费，坚决纠正损害群众利益的行为，打造阳光政府、廉洁政府。

      在看到成绩的同时，我们也清醒认识到，经济社会发展中还存在一些突出矛盾和问题。一是产业转型仍需加快。非煤产业总量偏小，服务业发展不足，战略性新兴产业规模不大，农业产业化水平不高。二是发展活力仍需增强。改革创新能力有待提升，经济外向度偏低，县区发展不平衡，园区综合实力有待提高。三是发展瓶颈仍需破解。土地、资金、环境等要素制约趋紧，通胀因素仍然存在，企业生产经营困难增多。四是发展环境仍需优化。节能减排和塌陷区综合治理压力加大，少数部门服务意识不强、行政效能不高，政府职能转变需要进一步加快。对此，我们将坚决采取措施，切实加以解决。

      各位代表，回顾一年来的工作，深感成绩来之不易。这是省委、省政府和市委坚强领导的结果，是市人大、市政协关心支持的结果，是全市人民奋发进取的结果。这里，我代表市人民政府，向全市人民，向驻淮部队、武警官兵和政法干警，向各民主党派、各人民团体和社会各界人士，向关心、支持淮南发展的同志们、朋友们，向在淮创业的境内外投资者、建设者，表示衷心地感谢！

      二、面向转型，明晰2012年总体思路

      今年是实施“十二五”规划承上启下的关键一年。我们既面临世界形势多变、国家调控趋紧、区域竞争加剧等诸多挑战，又面临中部崛起、沿淮开发、合肥经济圈提速等多重机遇；既面对结构调整、社会转型、环境约束等压力，又拥有资源丰沛、载体丰厚、项目丰硕等优势。能不能把握机遇、应对挑战，能不能争先进位、跨越转型，是对我们驾驭能力和执政能力的考验。成在发展，赢在转型。我们要凝心聚力、奋勇前行，奏响全面转型主旋律，打好富民强市攻坚战，在解放思想中打开视野，在加快转型中激发动力，在奋力赶超中争先进位，促进经济实力有新提升、产业结构有新优化、民生福祉有新水准、城乡面貌有新气象。

      今年政府工作的指导思想是：以邓小平理论和“三个代表”重要思想为指导，深入贯彻科学发展观，认真落实省、市第九次党代会精神，紧紧围绕科学发展、全面转型、富民强市，坚持“两型”引领，突出扩大内需，强化创新驱动，推进改革开放，着力普惠民生，确保稳中求进，实现包容发展，打造立体能源城、现代产业城、历史文化城、优秀旅游城、生态宜居城。

      今年经济社会发展的预期目标是：地区生产总值增长12%以上，财政收入增长13%以上，规模以上工业增加值增长15%以上，固定资产投资增长25%以上，社会消费品零售总额增长17%以上，城镇居民人均可支配收入、农民人均纯收入均增长14%，城镇登记失业率控制在4%以内，人口自然增长率控制在8.5‰以内，节能减排、安全生产完成省下达的目标任务。
在转型创新中实现经济社会平稳较快发展，是今年的首要任务。工作中，要突出把握四点：

      一是必须坚持稳中求进与全面转型有机统一。发展必须转型，转型也是发展。转型不仅有利于节约资源、保护环境，而且有利于培育和壮大新的增长点。我们既要在把握大势、发挥优势、保持态势中稳中求进，又要在应对挑战、化解矛盾、破解难题中加速转型。既要在全面转型的基础上求稳求进，又要在稳中求进的前提下全面转型，努力走出一条资源型城市可持续发展的新路。

      二是必须坚持“四煤”发展与“四宜”建设有机统一。全面转型的核心是产业转型。要着力构建节能增效现代产业体系，以“四煤”发展为突破口，在“立足煤”上推进清洁发展，在“延伸煤”上推进循环发展，在“不唯煤”上推进创新发展，在“超越煤”上推进优化发展。全面转型的关键是城市转型。要围绕打造宜居宜游宜学宜业城市，推进“1+6”空间布局，加快生态山南建设，实施滨高塘湖、滨淮河、滨凤凰湖、滨焦岗湖、滨瓦埠湖新区和北部新区开发，构筑生态安全屏障，把绿色优势转化为发展优势。

      三是必须坚持政府推动与市场调节有机统一。全面转型离不开政府推动和市场调节。要用好政府有形之手，强力实施“项目攻坚年”，推进项目、投资、招商“三位一体”突破，建立项目谈判、推进、督查三大机制，���除土地、征迁、融资三大困局。要用好市场无形之手，坚持科技创新、深化改革开放、完善体制机制，以科技引领发展，用好科学家资源；以产业支撑发展，用好企业家资源；以干部推动发展，用好政治家资源，营造一流的纳商、乐商、安商环境。

      四是必须坚持辛苦指数与幸福指数有机统一。推动全面转型必须保持想干事的激情、创新会干事的思路、增强能干事的魄力、提升不出事的境界，倍加珍惜大好机遇、大好势头、大好局面，真抓实干，务求实效。推动全面转型必须坚持以人为本，围绕“住有安居、学有优教、劳有多得、病有良医、老有颐养”目标，满腔热忱为民排忧解难，不遗余力保障改善民生，用干部辛苦指数提升群众幸福指数。

      三、迎难而上，推动又好又快发展

     （一）大力推进节能增效，切实转变经济发展方式

      坚持以“两型”发展提升产业素质，推动工业、服务业、农业“三箭齐发”，加快培育千亿产业、千亿企业、千亿园区。
推动工业向高新转变。着力培育煤电化三大产业和八大战略性新兴产业集群，推动淮南由平面能源城向立体能源城、传统能源城向现代能源城转变。全年完成工业投资260亿元，增长30%。新增规模以上工业企业50户。着力挖潜地下能源。推进朱集西矿建设，加快顾桥矿、潘三矿、张集矿技改，力争朱集矿、潘一矿东区投产。着力提升地面能源。开工建设淮沪二期、淮浙二期、平电三期、1000千伏特高压、2个500千伏变电站、凯迪生物质发电等项目，积极开展潘集电厂、洛电四期前期工作。着力拓展再生能源。加快日芯光伏一期投产、二期建设，开工建设毛集太阳能光伏发电项目。壮大化工产业。加快煤化一体化项目建设，开工建设煤制天然气，以及年产10万吨乙二醇试验装置，建成年产27万吨硝铵和稀硝酸生产线。壮大煤机装备制造业。支持比塞洛斯煤机、长壁煤机上水平，抓好凯盛重工、舜立机械、唐山开诚等30个项目，开工建设刮板输送机、液压支架等32个项目。壮大新能源汽车产业。确保陕汽新能源汽车投产，力争镍锌电池项目落户。壮大公共安全产业。发挥煤矿瓦斯国家治理工程研究中心等示范效应，促进公共安全技术产业化。培育信息技术产业。加快超声探测仪、激光雷达、“云海战略”创新实践区建设。培育节能环保产业。围绕节能减排、资源利用和环境治理，挖掘新的增长点。培育新材料产业。加快脱硫石膏板、新型墙体材料等产业发展。培育生物产业。推进医药生物园、国瑞药业工业园、药用辅料工业园和医药物流园建设，启动健康产业园规划。

      推动服务业向高端转变。出台加快十大服务业发展实施细则，建立健全服务业引导资金增长机制，启动省级服务业集聚区规划。支持服务业载体建设、环境优化，强化要素支撑、发展考核。做强现代商贸。推进东方国际购物中心、世贸商城、毛集文商城建设，开工建设山南城市综合体等项目，建成市体育商城。加快普及电子商务，精心筹办主题展销会和物资交流会。建设皖北物流结点城市。对接省现代物流发展规划，启动“两淮”物流中心和淮河流域外贸口岸基础设施建设。依托淮河岸线资源、芜湖港、河北港口集团，发展煤炭物流。提升淮矿物流、鑫森物流配送水平，加快粮食物流园、农产品加工物流园建设，培育第三方物流。健康发展房地产业。统筹商业、旅游、工业、科技地产开发，开工房屋面积300万平方米、竣工120万平方米。壮大金融业。编制金融业发展规划，出台加快金融业发展的实施意见，设立政府风险引导和产业发展基金。引进市外金融机构3家，开业小额贷款公司2家。力争金融机构各项贷款余额增长13%、中小企业贷款增长14%以上。健全科技服务业。加强研发设计、技术转移、科技创业和科技咨询等服务，提高瓦斯治理、电力检修等技术服务水平。提升信息服务业。强化信息技术和网络增值服务，加强物联网、云计算等技术应用开发。整合服务外包资源，支持服务外包企业开拓市场。拓展居民服务业。推进便利消费进社区、便民服务进家庭，扩大汽车、休闲、娱乐、健身等新型消费，推动咨询、评估、会计、法律等服务业发展，培育家政、养老和社区照料服务业。加强城市物业管理。深化农业服务业。规范农村综合服务社。推动“农机下乡”和“万村千乡”市场工程。新增“新网工程”配送中心2个。

      推动农业向高质转变。坚持以“两型”理念，推动优质高产高效和特色精品生态农业发展。一是粮食产业。落实国家粮食最低收购价等政策，创建稻麦“吨粮市”。抓好十大优质农产品生产基地、30万亩吨粮田、40万亩小麦高产攻关示范片、70万亩水稻提升行动核心示范区建设。二是蔬菜产业。加快四大蔬菜基地规划建设，全面完成标准化菜市场升级改造，提升“菜篮子”工程保障水平。三是畜牧业。加大对养殖大户扶持力度，推进标准化养殖场建设。加强与泰国正大集团等合作，扩大肉制品、奶制品生产。四是水产业。打造沿淮沿湖优质水产板块，探索采煤塌陷区立体生态养殖模式。新增生态网箱养殖1万立方米，完成退网还湖4.2万亩。五是豆制品产业。发挥豆腐故里的品牌效应，规划建设豆制品加工、销售、美食一条街。六是特色农业。支持黄金梨、草莓、食用菌、淮王鱼、农家乐等特色农业发展，提升规模化、标准化、产业化水平。七是农产品加工业。以粮食产业化“60亿跨越工程”为龙头，加快凤台粮食工业园等建设。新增市级以上龙头企业10家、专业合作社50家。新建市级以上农业标准化示范区10个，认证“三品”10个以上。八是发展劳务产业。强化“三农”职业技能培训，鼓励农民就地就近创业就业。全年培训新型农民1.5万人。

      （二）大力推进生产力布局调整，提高城矿乡统筹水平

      以“两型”标准发展园区，统筹现代农业示范园建设、工业开发区扩容升级、采煤塌陷区综合治理，推动布局集中、产业集聚、用地集约，凸显园区生态、低碳、循环特色。

      启动现代农业示范园建设。创新“1+6”布局，推进毛集整区开发和6个县区示范园建设。坚持以科学、市场和工业的理念，加快高标准规划、高水平配套、高起点开发、高强度招商，构建“园中园”。落实开展现代农业示范园建设意见，设立5000万元引导资金，整合各类涉农资金，支持基础设施、集中居住区和重点产业项目建设。出台加快土地流转的政策意见，建立县区土地流转中心、仲裁机构和村级服务平台，促进连片开发，形成规模效应。

      加快工业开发区扩容升级。完善“4+7”布局，提升市里主抓的4个、县区主抓的7个园区功能。开工建设标准化厂房30万平方米以上。完成企业退城进园三年计划。力争市经济技术开发区创建国家级开发区。完善东部工业区路网和配套设施，启动农场棚户区和东方瑞景等老城改造项目。推进浙商产业园、明发城市综合体等16个项目，加快三一重工等18个项目建设，确保北新建材等14个项目投产，争取主要经济指标增长20%以上。夯实争创国家级高新区基础。进一步完善高新区服务功能,出台加快高新区发展意见，完善高新区扩展规划。推进煤化工产业园建设。开工建设煤制甲醇及转制烯烃主体工程，有序推进综合利用发电项目。启动铁路专用线、污水处理厂等配套建设。加快淮南集中区规划。完善园区共建共享利益分配机制。

      实施采煤塌陷区综合治理。统筹“1+4”板块，加大潘谢新矿区综合治理，推进毛集、八公山、谢家集、大通等4个老矿区生态修复。加快集中式搬迁、发展式安置、开发式治理，统筹解决就业、就学、就医等生产生活问题。新开工搬迁安置点10个、竣工7个，确保凤凰湖、瓦谢新村、潘东新城一期等5个安置点搬迁入住。大力推进采煤塌陷区综合治理项目库和彭苏萍院士工作站建设，继续推广“凤凰湖”、“后湖”、“鑫森”、“绿馨园”等综合治理模式，变采煤塌陷区为开发区、生态区和希望区。

      （三）大力推进“两型”设施发展，打造生态宜居城市

      把“两型”理念贯穿于城市规划、建设、管理全过程，实施高起点规划、高品质建设、精细化管理。全年完成大建设投资350亿元。

      用“两型”理念提升城乡规划。按照立体发展、集约建设和生态宜居的要求，强化规划调控作用。坚持“四规合一”，形成城乡一体、配套衔接、全面覆盖、支撑有力的规划体系。编制主城区20平方公里控制性详细规划，完成城市地下空间开发利用等8个专项规划、2个村镇总体规划。加强城乡规划落实、管理，注重从源头上控制征地、拆迁、绿化等问题，建立规划考评体系。
用“两型”标准建设现代城市。牢固树立绿色建设理念，打造“山清、水秀、天蓝、景美”的生态城市。启动滨水开发。加快滨高塘湖新区站前路、滨湖大道建设，完成站前广场、朝阳东路延伸段等工程，启动大通政务新区规划，形成“高铁”效应，提升东部形象。加快滨淮河新区规划设计，统筹推进起步区基础设施建设和淮河岸线资源开发，开工建设田家庵政务新区。推进滨凤凰湖新区、滨焦岗湖新区综合性开发，开展滨瓦埠湖新区规划设计。建设生态山南。全年续建项目78个，新开工项目39个，完成投资100亿元以上。确保市级政务中心6月份搬迁投用，基本建成中央水系公园、奥体公园主体育场。加快供电、供气和污水处理等设施建设，开工建设淮师附小、实验中学、五星级酒店、哈米王国儿童体验中心、物联网小镇等项目。支持淮南矿业推进山南新区13.6平方公里开发。加快老城改造。按照“成片改造、综合提升、一体推进”原则，启动黎明东村等10个综合改造项目。实施龙王沟等水系改造，落实城市水系综合改造亚行贷款项目。完成人防工程7万平方米。

      高标准建设新农村和治淮工程。坚持以农村危旧房改造和村庄整治、塌陷区村庄搬迁、新市镇建设为抓手，加快城乡一体化示范村镇建设。整村推进10个村扶贫开发。农村清洁工程扩大到500个村。完成危桥改造20座、县乡道路升级改造8条，推进村级道路和农村电网改造。争取西淝河等洼地治理应急工程批复实施，加快焦岗湖圩堤、凤凰湖排涝站、架河节制闸等项目建设，完成小型水库除险加固4座。解决农村8万人饮水安全问题。加大农田水利基本建设投入，提高防汛减灾水平。

      积极构筑综合交通。树立“全域”交通理念，构建内部成网、外部成环、内畅外联的交通体系。建成京福高铁淮南段、淮蚌高速公路和10条道路续建工程。加快孔李淮河大桥、凤台二桥、102省道、308省道等项目建设。支持济祁高速、商杭高铁淮南段等建设，开工建设九龙大道、十涧湖东路和专用码头等项目，做好淮河隧道开工准备。抓好合淮城际轻轨、淮滁高速公路、西部南北通道、城市快速路网前期工作。

      （四）大力推进科技创新，构筑“两型”发展新优势

      坚持科技引领，让更多创新要素在淮南集聚、更多创新成果在淮南转化、更多创新企业在淮南发展，为“两型”城市建设提供科技支撑。

      集聚创新要素。大力实施全民科学素质行动计划纲要。以国家技术创新工程试点省建设为契机，建设创新型城市。加强洁净煤、太阳能光伏等技术研究，构建产学研联盟。加快煤炭开采国家工程技术研究院建设，筹建国家级药用辅料研究中心、省煤电化节能检测中心、省煤矿安全仪器设备检测中心。加快中科院合肥物质研究院等7个研发中心建设。积极培育中国名牌产品。申报中国驰名商标1件，争创省著名商标15件。制订国家、行业标准2个。

      转化创新成果。以高新技术产业开发区为载体，推进台商科技园、科技创新公共服务中心等建设，促进美国安科光纤通讯器材、清华同方数码产业园等项目落地，推动无人机、ET超导、溶岩储能项目产业化。启动科技孵化楼建设。申请专利1700件，授权700件。申报高新技术企业15家、高新技术产品60个、省级创新型试点企业2家、省级工程技术研究中心3家。高新技术产业增加值增长20%以上。

      培育创新企业。出台发展总部经济的政策措施，吸引世界一流技术、世界五百强、国内五百强企业落户淮南。落实“千百十”工程实施意见，培育超50亿元企业、超10亿元企业各2户。用足用活科技创新政策，探索企业股权激励分红政策试点，推动优势企业争当行业排头兵。加大技改力度，实施200个技改项目。推进信息化与工业化深度融合，建设数字企业100户，申报省“两化融合”企业5户。实施小型微型企业成长工程，突出化解要素制约。建立市级中小企业服务平台，引导企业做好市场开拓和产销衔接。扶持20户专精特新、30户成长性企业做大做强。支持民营企业多元发展，新增民营企业1200户，非公经济增加值增长18%以上。

      （五）大力推进改革开放，激发“两型”发展活力

      坚持把改革开放作为“两型”建设的活力源泉，坚决破除阻碍“两型”发展的体制机制，拓展发展空间。

      提升招商势头。坚持产城一体、筑巢引凤、资源共享、错位互补，优化投资环境，提高招商实效。深化与合肥经济圈能源、交通、产业和招商合作，探索合作园区共建和集群式承接新模式。建立重大项目签报制度，创新地块招商、楼宇招商、产业招商和驻点招商。做实节庆招商，提升中国豆腐文化节规格。抢抓新一轮产业转移机遇，瞄准长三角、珠三角、欧美等区域，定向推介。选准一批央企、省企、民企、外企，定向跟踪。对准集中开工、在谈招商项目，定向突破。确保全年引进内资增长25%以上、利用外资增长12%以上。支持出口企业加快外贸转型，实现进出口总额增长10%以上。加强对外交流，建立友好城市1个。
拓展改革源头。建立健全“两型”体制机制和考核标准，成立“两型”办。深化行政管理体制改革，开展事业单位分类改革。完成2户国企改革，实现企业上市1户，新增后备上市企业6户。完善资源开发治理生态补偿机制、资源性产品价格形成机制。深化招投标监管体制改革，进一步规范招投标市场秩序。建立土地征收保障考核机制，强化县区征收主体责任。完善用地保障机制，实施土地“双保工程”，整理土地0.8万亩。建立财政金融撬动发展机制，筹建市金融控股公司，启动山南金融集中区建设。推动通商银行、淮河银行、小额贷款公司和融资性担保公司增资扩股，推进凤台联社改制。五大投资公司完成融资70亿元以上。

      增强县区劲头。落实支持县区发展的各项政策，提升跨越转型能力。支持凤台推进深煤非煤发展、老城改造和新区建设，打造淮河明珠。支持田家庵扩张二产、提升三产、做精一产。支持大通发挥综合优势，建设特色工业基地、绿色农产品供应���地、商贸物流基地、宜居宜游基地。支持谢家集推进工业强区、商贸大区、农业特区、生态矿区建设。支持八公山旅城、产城、城乡一体发展。支持潘集发挥煤电化主战场优势，推进新型工业化、新型城镇化、城乡一体化。支持毛集国家可持续发展先进示范区建设，推动旅游区、中心区、开发区互动，创建国家级生态示范区。推进6个省级产业集群镇发展，落实扩权强镇试点政策。实施民族乡村“优先发展提升行动”，实现特色发展。

        （六）大力推进文化强市，彰显“两型”文化特色

      “两型”文化，是五彩淮南的精神食粮。必须坚定文化自觉，增强文化自信，推动文化繁荣。

      树立文化信仰。坚持用社会主义核心价值体系引领风尚、提振精神、塑造形象，推进社会公德、职业道德、家庭美德、个人品德建设。加强全社会信仰教育，弘扬新风正气，崇尚伦理道德，塑造城市精神，夯实共同奋斗的思想基础。积极推广“两型”文化，创建一批“两型”学校、机关、企业。强化诚信文化，制定社会信用体系建设指导意见。提炼淮南文化元素，设计“五彩淮南”形象标识。

      发展文化产业。出台淮南文化大发展大繁荣实施意见，制定文化产业发展规划。启动文化产业振兴工程。依托特色文化资源，放大节庆品牌效应，打造一批文化产业基地。申报国家文化产业示范基地2家。积极发展印刷、娱乐等传统文化产业，壮大文化创意、动漫游戏、数字出版、网络视听等新型文化产业。编制文化企业名录，培育一批文化骨干企业。文化产业产值增长50%以上。加快文化旅游融合。推进“千亿文化旅游振兴工程”。开工建设新四军文化园、春申君战国文化园等10个项目，加快八公山旅游综合服务区等一批项目建设，建成焦岗湖度假村、八公山地质遗迹保护等10个项目，确保泰坦方舟动漫园开园。新增国家4A级景区1处。全年接待游客突破1000万人次，实现旅游收入45亿元以上。

      繁荣文化事业。实施文化惠民工程，加快淮南文化艺术中心建设，推动市博物馆新馆、日军侵华罪行遗址纪念馆、淮南煤炭博物馆规划建设。积极申报国家公共文化服务体系示范区，加大示范项目推进力度。加快凤台花鼓灯艺校、基层文化活动中心建设，建成农家书屋和社区书屋160个。出版发行淮南市志等书刊。完成有线电视数字化改造4万户。精心组织“大地欢歌”等文化活动。完善文化事业单位内部职能，创新广播电视、文化市场管理，构建现代文化市场体系。

      推进“五城联创”。建立健全工作机制，不断提升各项创建水平。启动国家环保模范城创建。精心编制创建规划，制定创建指标体系，谋划一批创建项目，夯实创建基础。创建全国绿化模范城。全年成片造林1.3万亩，义务植树400万株。建成绿化模范乡镇、村20个，新建绿色小康村25个。确保顺利通过验收。争创国家生态园林城。续建十涧湖公园一期、舜耕山风景区，开工建设淮西湖公园一期、淮河公园。加大“绿色图章”执行力度，新增城市绿地100万平方米。创建省级以上生态乡镇2个。争创全国卫生城。对照国家卫生城市标准体系，扎实推进城乡卫生基础设施建设。创建省级卫生县城、卫生乡镇各1个。争创全国文明城。制定创建规划和年度计划，建立目标管理责任制和综合考评体系。加强市容环境综合整治，推进城乡生活垃圾资源化、无害化、减量化处置，完善长效治理机制。整治小街小巷30条。精心组织“讲文明、树新风”系列活动。推进民族团结与文明创建有机结合。成功创建省级文明城市。

       （七）大力推进民生改善，提高群众幸福指数

      民生连着民心，民心凝聚民力。建设“两型”城市，目的是创造人民群众更加幸福美好生活。

      实施民生普惠工程。深入落实民生工程“十二五”规划，建立健全长效惠民机制。全年投入15亿元以上，实施35项民生工程。加快保障性住房建设。严格落实保障性住房配建制度，出台保障性住房权属、供给和管理机制。新建廉租住房1132套、公共租赁住房5476套、棚户区改造住房5508套。提高群众健康水平。深化医药卫生体制改革，推进公立医院综合改革试点。开工建设市精神卫生中心，改扩建县级保健所7个、县级监督所6个。提高新型农村合作医疗个人筹资比例和补偿标准，实现城镇基本医疗保险市级统筹。建成农民体育健身工程50个、乡镇文化体育站10个。大力实施“宜家工程”。开工建设人口和家庭公共服务中心，提高计生家庭发展能力。完善社会保障体系。扩大社会保险覆盖面。建立健全社会化养老机制。出台城乡居民养老保险意见，实现养老保险全覆盖。农村五保集中供养率达50%。完善城乡低保标准自然增长机制。完成市社会福利保障区及配套建设。加强物价调控监管，保持总水平基本稳定。

      实施素质提升工程。把办好教育作为提升素质、促进就业的基础。全面实施学前教育三年行动计划，新建21所、改扩建7所普惠性幼儿园。推进义务教育均衡发展，确保2个区实现省级义务教育基本均衡发展目标，创建省级义务教育经费保障机制改革示范县区2个，提高农村学校标准化建设水平。加快安徽理工大学新校区建设。确保淮南二中新校区秋季投用、淮南体育学校秋季招生。开工建设市职教园区和淮南技师学院，支持淮南联大开展中职高职互联试点，启动淮南保安学院规划，积极筹建淮南少儿艺术学院。关心支持特殊教育。加快人力资源市场、公共就业实训基地、培训平台建设。建立企业用工和失地农民就业联动机制。新增城镇就业岗位3万个、下岗失业人员再就业1.8万人。

      实施创业富民工程。成功创建省级创业型城市，全力争创国家级创业型城市。完善创业服务体系，加大创新创业人才队伍建设。发放小额担保贷款1.2亿元。新建各类创业园6个，申报留学人员创业园1个、省级引智基地1个、企业博士后科研工作站2个。继续实施“人才青苗工程”，引进和培养一批创业型领军人才。

      实施财富增值工程。坚持科学理财，大力培植财源。实施“阳光财政”，强化财税征管。加大财产权益保护力度，建立健全工资正常增长机制。实施居民收入倍增规划，增加农民、企业职工、中低收入者和困难家庭收入，提高居民工资性、经营性、财产性、转移性收入，让城乡居民收入年年都有新增加、生活年年都有新改善、面貌年年都有新提升。

      （八）大力推进管理创新，维护社会和谐稳定

      管理创新是建设“两型”城市的根本保障。既要通过管理创新提升发展质量，更要通过管理创新提升生态环境，努力打造平安淮南。

      控好污染源。加快申报国家再生资源回收体系建设试点城市。加强重点企业用能管理，实施节能与资源综合利用项目30个。启动矿产资源综合治理，同步推进污染防治、资源整合、土地回收、生态修复。加快建设潘集、凤台污水处理设施，开工建设八公山、毛集污水处理厂和7座生态式污水处理站，推进城市雨污分流截流等管网工程。新建大中型沼气池1处、户用沼气池1000口。全面推行机动车排气环保标志管理制度，大力实施火电机组脱硝工程。开展农村环境连片整治，加强畜禽养殖场等面源污染治理。

      织好管理网。加快数字城管二期工程建设，完善大城管格局。实施交通畅通工程，调整和优化交通线路，加强公共交通规范化管理。加大社区居委会建设力度，夯实管理基础。全面推行网格化管理，建立生产生活难题、热点难点问题、便民惠民举措服务网络，提高网格化管理的组织效能、运行效率和社会效益。

      建好平安城。加强公共安全体系建设。严格落实安全生产各项制度，提高防范重特大事故的能力。完善疾病预防控制、食品药品监管责任体系，防范公共卫生和食品药品安全事件发生。建成国家矿山应急救援淮南基地，筹建市级减灾救灾指挥中心和救灾物资储备库。健全突发事件应急预案体系，推进应急管理体制、机制、法制建设，提高重大突发事件处置能力。

      架好连心桥。深入开展大走访、大接访等活动，扎实推进“政策攻关”，弥合利益分歧、扩大社会共识、凝聚群众力量。真心关爱妇女儿童、残疾人和老龄工作，切实做好外事、侨务、优抚安置、国家安全和双拥共建等工作。深入开展“六五”普法，推进依法治市。提高法律援助规范化水平，健全人民调解、行政调解、司法调解联动机制。加强公安机关职业素养和能力素质建设，提高专业化、职业化水平。坚持重大事项社会稳定风险评估，完善矛盾纠纷预警机制，努力从源头上化解和减少矛盾。健全维稳综治数字化、信息化防控体系，构建全方位、全天候、立体化防控网络，增强群众安全感和幸福感。

      四、奋发有为，提升勤政廉政形象

      各位代表，优势总在抢抓机遇中形成，差距常在错失机遇中扩大。我们必须倍加珍惜发展机遇，勇于承担历史使命，奋力推进“两型”城市建设。

      要善于凝聚“两型”发展的智慧力量。“两型”发展，是重大课题，必须站得高、看得远、谋得深。要坚持科学决策。深入开展调查研究，既解剖典型又掌握全局，既总结经验又正视矛盾。要加强理论联系实际，从战略高度、全局角度、谋划深度上把握，加快宏观政策项目化、上级精神淮南化、外地经验本地化、原则规定具体化。要坚持民主决策。自觉向群众、向基层、向实践学习，通过民情民意调查、建议建言征集、参政议政平台，扩大公民有序参与决策渠道。要坚持依法决策。深化行政决策机制示范市建设，健全行政决策合法性审查机制，打造法治政府。

      要勇于担当“两型”发展的历史使命。大力弘扬求真务实的实干精神、争分夺秒的抢先精神、迎难而上的攻坚精神，做到既谋战略、又谋战术，既会出题、又会破题，既有想法、更有办法。要把目标任务落实到具体行动中，定下的事要迅速执行到位，看准的事要坚决实施到位，困难的事要切实解决到位。要进一步优化审批环境、政务环境、生态环境，推行网上审批，杜绝不作为、慢作为、乱作为等现象。要落实“一岗双责”，加强明查暗访，严查不落实的事，处理不落实的人，解决不落实的问题，靠实干说话、凭实绩用人、用实效检验。

      要勤于修炼执政为民的服务本领。把群众工作贯穿于“两型”城市建设的全过程，在思想上尊重群众、感情上贴近群众、工作上依靠群众。要加强网络问政和在线交流，既要上网收集网情，又要下网办好网事。要加强政务公开，扩大群众的知情权和参与权。要深入群众、深入基层、深入一线，帮助群众解难事、破难题、度难关，坚决查处影响群众工作、损害群众利益的行为。
要严于执行廉洁从政的制度要求。认真落实市委决策部署，打造思想共识、情感共融、工作共进的政府班子。自觉接受人大、政协、人民群众和新闻媒体的监督，认真办理人大代表建议和政协委员提案。要深入推进反腐倡廉，开工建设市党风廉政教育中心，全面推行公务卡制度，严格控制“三公”支出，把有限的资金用在发展经济、改善民生上。要加强对领导干部、关键岗位，以及人财物使用管理的监督和制约，严格执行行政监察和审计监督，防止权力失控、决策失误、行为失范，确保政务规范、政事清明、政风端正。

      各位代表，新的年轮开启新的希望，新的征程承载新的使命。让我们在市委的坚强领导下，团结带领全市人民，抢抓机遇，坚定信心，应对挑战，破浪前行，努力在“两型”城市建设的伟大实践中实现五彩淮南新跨越！
